# Negative Impacts of Self-Stigma on the Quality of Life of Patients in Methadone Maintenance Treatment: The Mediated Roles of Psychological Distress and Social Functioning

**DOI:** 10.3390/ijerph16071299

**Published:** 2019-04-11

**Authors:** Ching-Ming Cheng, Chih-Cheng Chang, Jung-Der Wang, Kun-Chia Chang, Shuo-Yen Ting, Chung-Ying Lin

**Affiliations:** 1 Jianan Psychiatric Center, Ministry of Health and Welfare, No. 80, Ln. 870, Zhongshan Rd., Rende Dist., Tainan 71742, Taiwan; babyming@gmail.com (C.-M.C.); kunchiachang0517@gmail.com (K.-C.C.); 2 Department of Natural Biotechnology, College of Science and Technology, Nanhua University, Chiayi 62249, Taiwan; 3 Department of Psychiatry, Chi Mei Medical Center, Tainan 70246, Taiwan; rabiata@gmail.com; 4 Department of Health Psychology, Chang Jung Christian University, Tainan 71101, Taiwan; 5 Centre for Global Mental Health, Health Services and Population Research Department, Institute of Psychiatry, Psychology and Neuroscience, King’s College London, London SE5 8AF, UK; 6 Departments of Internal Medicine and Occupational and Environmental Medicine, National Cheng Kung University Hospital, College of Medicine, National Cheng Kung University, Tainan 70101, Taiwan; jdwang121@gmail.com; 7 Department of Public Health, College of Medicine, National Cheng Kung University, 1 University Road, Tainan 70101, Taiwan; 8 Chang-Hua Hospital, Ministry of Health and Welfare, Puxin Township, Changhua County, Puhsin 51341, Taiwan; kamating@yahoo.com.tw; 9 Department of Rehabilitation Sciences, Faculty of Health and Social Sciences, The Hong Kong Polytechnic University, Hung Hom, Kowloon, Hong Kong

**Keywords:** self-stigma, opioid, quality of life, structural equation modeling

## Abstract

A sample of heroin users (*n* = 250) in methadone maintenance treatment (MMT) was used in this cross-sectional study to clarify the mechanisms of the effects of stigma on quality of life (QoL) through psychological distress and social functioning. All the participants had their self-stigma, psychological distress, social functioning, and QoL measured. Psychological distress and social functioning were proposed to be mediators between self-stigma and QoL. Several linear models using structural equation modeling were conducted to examine the mediated effects. The negative effects of self-stigma on QoL were significantly mediated by psychological distress, as self-stigma directly and significantly influenced psychological distress, but not social functioning. This study demonstrated a linear model describing the effects of self-stigma on QoL for opioid-dependent individuals; psychological distress was also an important mediator between self-stigma and their QoL. Clinicians were able to notice the importance of reducing self-stigma for opioid-dependent individuals according to the following results: higher levels of self-stigma were associated with high psychological distress, decreased social functioning, and impaired QoL. Our mediation findings suggest that treating psychological distress is better than treating social functioning if we want to eliminate the effects of self-stigma on QoL for heroin users.

## 1. Introduction

Research and discussion on stigma grew substantially after Goffman [1] (p. 3) defined stigma as an “attribute that is deeply discrediting.” Since then, different types of stigma have been defined according to different systems. For example, Livingston and Boyd [2] used three hierarchical levels, including the system or macro level (structural stigma), the group or meso level (public stigma), and the individual or micro level (self-stigma or internalized stigma) to classify stigma. Brohan et al. [3] and Corrigan and Rao [4] used awareness, experience, and acceptance to define different types of stigma as perceived stigma, experienced stigma, and self-stigma. Some researchers have also discussed the stigma of the friends and relatives of the stigmatized population, such as courtesy stigma [1], associative stigma [5], and affiliate stigma [6,7]. Moreover, Link and Phelan proposed the underlying mechanism of the stigma using the co-occurrence components: labeling, stereotyping, separation, status loss, and discrimination [8]. Furthermore, a trend toward in-depth discussion of self-stigma is developing because more and more studies have confirmed its importance [9,10,11,12,13].

Self-stigma, the self-devaluation and acceptance of public stigma, serves as a barrier to the pursuit of valued life goals [14]. However, little attention has been paid to the self-stigma related to substance use disorders [15,16] even though many studies have investigated the issue for other psychiatric disorders [9,11,17,18]. To the best of our knowledge, current research on self-stigma among substance users focuses on instrument development [16] and self-stigma reduction intervention [15,19]. Although the two aforementioned topics are crucial for mental health professionals, there is a need to investigate the possible mechanism by which self-stigma correlates with overall health outcomes, such as quality of life (QoL), for substance users. Knowing the mechanism will provide healthcare providers with insightful knowledge to treat the substance users immediately and appropriately [20]. For example, if we find a significant mediator for the effects of self-stigma on QoL for substance users, the treatment can be designed on the mediator in addition to reducing self-stigma. With the literature showing that substance users have problems in psychological distress and social dysfunction [21,22], two dominant factors in QoL, we proposed that psychological distress and social functioning are two important mediators for the effects of self-stigma on QoL. Additionally, important confounders, including age, gender, educational level, and Hepatitis C virus (one of the most common infectious diseases for substance users), should be considered in the proposed mechanism.

QoL is an important health index in mental health service delivery due to the paradigm shift of recovery; that is, the focus for people with mental illness changes from reliving symptoms to improving QoL [23]. Therefore, QoL among people with mental illness has been widely studied. Indeed, the negative effects of self-stigma on QoL have been found in people with severe mental illness [24,25], depressive disorders [18], and substance use [22]. However, we need to further explore the underlying factors between self-stigma and QoL. That is, we need to further understand to what extent self-stigma impacts substance users and the possible routes between self-stigma and QoL. In addition, we proposed that psychological distress and social functioning could be two possible mediating factors between self-stigma and QoL. The effects of psychological symptoms on the QoL have been found [26,27,28,29]; that is, psychological distress of an individual is very likely to influence his/her QoL. Given that self-stigma is a potential dominator of psychological distress [17,30], it is reasonable to hypothesize that a mediated effect exists between self-stigma and QoL for individuals, including substance users.

As for social functioning, people with mental illness who have a higher level of self-stigma tend to have poorer social functioning [31]. A relationship between self-stigma and social functioning was also found in 34 people with first-episode psychosis although the relationship was diminished after controlling for symptom severity [32]. Moreover, an association between impaired social functioning and poor QoL was found in a Dutch population of psychiatric outpatients [33]. Therefore, social functioning could be another mediator between self-stigma and QoL in addition to psychological distress. Also, psychological distress is associated with social functioning [34], and we thus hypothesized that psychological distress could affect the social functioning of substance users. Therefore, social functioning could serve as another mediating factor between self-stigma and psychological distress.

Although the relationships between self-stigma, psychological distress, social functioning, and QoL have been well-documented [30,33,34,35,36], to the best of our knowledge, no studies have discussed in-depth any mediating models using these four factors. How self-stigma associates with health should be discussed, especially among heroin users in Taiwan. According to the law regarding illicit drug use, heroin users were viewed as criminals [37]. It was not until 1998 that the Drug Control Act revised the legal identification of them as both criminals and patients [38]. Nevertheless, a systematic review reveals that healthcare providers may have negative opinions toward heroin users because heroin users may overuse the resources, are not vested in their own health, and have poor adherence to recommended care [15]. That is, even though some heroin users have satisfactory adherence, healthcare providers still perceive them as not responsible to their treatment. Similarly, Stuart [39] recently summarized that public and healthcare providers perceive “medical therapy,” “medication substitution therapy,” and “prescribed opioid” as a character weakness. With the stigmatization, heroin users are likely to refuse to engage in services. Accordingly, the health, including psychological health and social relationships, of heroin users is jeopardized. Although Taiwan government introduced the methadone maintenance treatment (MMT) programs in 2005 to solve the urge for inhibition for the spread of HIV infection among injective heroin users [40], only infected patients were prioritized for treatment. Additionally, those receiving MMT may further develop self-stigma and subsequently have impaired health. 

Because most of the studies discussing the mechanisms regarding self-stigma have used samples with severe mental illness, especially schizophrenia, there is a gap in terms of substance users in the literature. Therefore, this study was aimed toward an investigation of the relationships among the aforementioned factors (self-stigma, psychological distress, social functioning, and QoL) in treated opioid-dependent individuals. Specifically, we hypothesized that self-stigma, psychological distress, and social functioning are associated with QoL; psychological distress and social functioning are mediators between self-stigma and QoL; social functioning is a mediator between self-stigma and psychological distress.

## 2. Materials and Methods

This study was approved by the Institutional Review Boards of the Jianan Psychiatric Center (JPC14-022) and the Chi Mei Medical Center, Tainan, Taiwan (10403-004).

### 2.1. Participants

Heroin users registered into MMT programs were recruited from three hospitals that had the largest population with heroin addiction in central and southern Taiwan between April 2015 and February 2016. The three hospitals are the largest long-term opioid agonist treatment sites in central and southern Taiwan. Several psychiatrists determined the eligibility of the participants based on the following inclusion criteria: (1) aged 20 years or older; (2) meeting the DSM-IV (Fourth edition of Diagnostic and Statistical Manual of Mental Disorders) criteria for opioid dependence; (3) no other MMT contraindication, such as severe liver disease or acute psychosis; and (4) having sufficient mental capacity to provide informed consent and complete the assessment.

After receiving the signed written informed consents of the eligible participants, data were collected using self-administered questionnaires and interviews with a research assistant (or case manager). The research assistants and/or case manager from each hospital received a half-day training course to ensure the inter-rater reliability of the Opiate Treatment Index (OTI). The participants analyzed had characteristics similar to those of Taiwanese nationally representative data [41]. 

### 2.2. Measures

The instruments used in this study included a demographic questionnaire and clinical records, the Self-Stigma Scale-Short (SSS-S), the OTI, and the World Health Organization Quality of Life-Brief version (WHOQOL-BREF). The demographic questionnaire and clinical records were used to collect information regarding age, marital and living status, educational attainment, employment and income status, HIV and hepatitis status, use of medications, and substance use history as well as the previous episodes and duration of MMT.

#### 2.2.1. Self-Stigma Scale-Short

The SSS-S consists of 9 self-stigma-related items in three dimensions, namely, cognition, affect, and behavior, with 3 items in each [13]. Each self-rated item is rated on a 4-point Likert scale ranging from 1 (strongly disagree) to 4 (strongly agree). Because the SSS-S is designed for different minority groups, the term for the minority group can be replaced with the group being tested, and it has been applied fairly for “mental illness” groups in Taiwan with satisfactory Cronbach’s alpha (0.95) [42]. In this study, we used the term “heroin users” instead of “methadone patients” to specify our study population in the SSS-S and make it more understandable to the participants. Moreover, we used the average score of the nine items to present the level of self-stigma. The Cronbach’s alpha of the SSS-S in the current study is 0.89.

#### 2.2.2. Opiate Treatment Index for Psychological Distress and Social Functioning

The Opiate Treatment Index (OTI) consists of 6 domains: drug use, HIV risk-taking behavior, social functioning, health status, and criminality and psychological adjustment (distress) over the past one month preceding the assessment interview, with the exception of the social functioning scale covering the preceding 6 months [43]. A higher score in a domain indicated more severe dysfunction for that particular domain. In this study, we only adopted two domains that were relevant to our study aims: social functioning and psychological adjustment. The social functioning domain comprised 12 questions with a 5-point Likert scale on aspects of social integration, such as employment, residential stability, and interpersonal conflicts. Therefore, the score range of the social functioning domain was between 0 and 48, where 0 indicates the best and 48 indicating the worst functioning. 

In the original OTI English version, the items for the psychological adjustment domain were adopted from the General Health Questionnaire-28 (GHQ-28) [44]. In the same domain of the OTI Chinese version, there are 12 items with a 4-point Likert scale (scored 0–3) from the Chinese Health Questionnaire (CHQ-12) [45], which was revised from the GHQ-28. The CHQ-12 (range: 0–36) was adopted because of its stronger psychometric properties compared with the GHQ-28 in a Taiwanese context [45,46]. A higher score in the psychological adjustment domain indicates more psychological distress. 

The OTI has been found to be a valid and reliable instrument for outcome evaluation of addiction treatment in Taiwan [47]. The values of the inter-rater reliability and content validity of the Chinese version of OTI were good: the kappa values and Cronbach’s alpha were 0.706 and 0.663 for the domain of social functioning and 0.680 and 0.871 for psychological adjustment, respectively [46]. The Cronbach’s alpha values were 0.64 for the social functioning and 0.88 for the psychological adjustment in the current study.

#### 2.2.3. The World Health Organization Quality of Life-Brief Version

The WHOQOL-BREF Taiwan version measures QoL using 26 items from the original WHOQOL-BREF and 2 Taiwanese national items [48]. The WHOQOL-BREF Taiwan version consists of four domains, namely, physical (7 items), psychological (6 items), social relationships (4 items), and environment (9 items), with 2 generic items measuring overall QoL and health. The item scores were rated on a five-point scale, and each domain score ranged from 4 to 20 points, with a higher score indicating a better QoL. In addition, satisfactory psychometric properties have been established for the WHOQOL-BREF Taiwan version: Cronbach’s alpha values were between 0.70 and 0.91 [48,49]. Moreover, the Cronbach’s alpha of the WHOQOL-BREF Taiwan version in the current study is 0.93.

### 2.3. Statistical Analysis

All the statistical analyses were performed using SPSS 17.0 (SPSS Inc., Chicago, IL, USA), except for the linear structural equation modeling (SEM) using AMOS 7.0 (SPSS Inc., Chicago, IL, USA). 

Demographics and clinical characteristics were analyzed using descriptive statistics: continuous variables with mean and SD, and categorical variables with frequency and percentage. In addition, we examined how many participants had a high level of self-stigma using a cutoff of 2.5 [9]. Specifically, the score of 2 indicates “agree on the description of self-stigma,” and thus, a score higher than 2 (where we used 2.5) suggests a high level of self-stigma.

**Figure 1:**
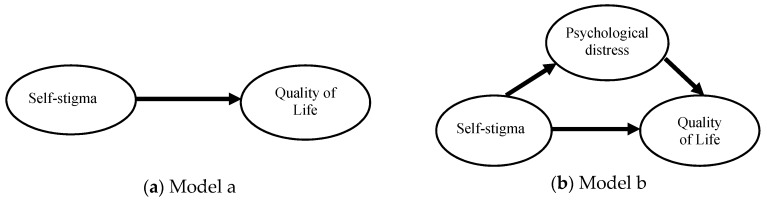

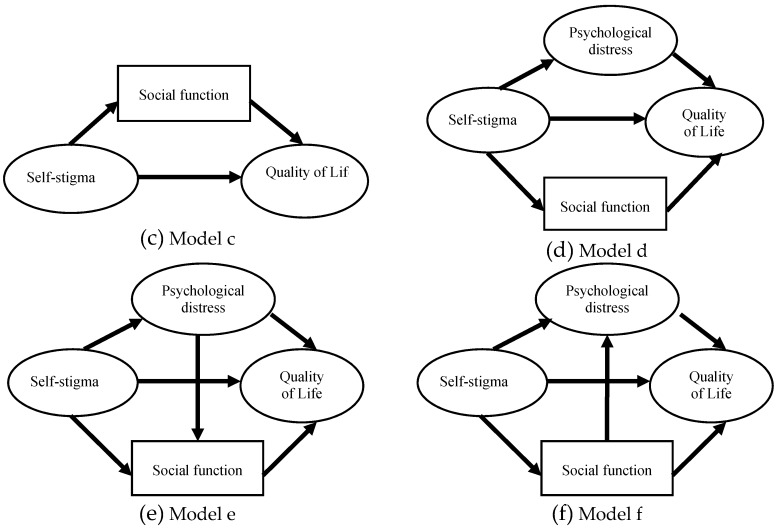
Proposed paths between self-stigma, quality of life (QoL), psychological distress, and social function. (**a**) Total effect of self-stigma on QoL; (**b**) Mediated effect of self-stigma on QoL via psychological distress; (**c**) Mediated effect of self-stigma on QoL via social function; (**d**) Mediated effect of self-stigma on QoL via psychological distress and social function; (**e**) Mediated effects of self-stigma on QoL via psychological distress, social function, and psychological distress through social function; (**f**) Mediated effects of self-stigma on QoL via social function, psychological distress, and psychological distress through social function.

The proposed mechanisms were analyzed using linear SEM [50], and a total of 6 linear models were examined. Model a (Figure 1a) investigated the total effect of self-stigma on QoL. Models b (Figure 1b) and c (Figure 1c) included the mediated effects of self-stigma on QoL via psychological distress and social functioning, respectively. Models d (Figure 1d), e (Figure 1e), and f (Figure 1f) simultaneously took the mediated effects of self-stigma on QoL via psychological distress and social functioning, while Models e and f additionally considered the association between psychological distress and social functioning in different directions (Model e is psychological distress on social functioning; Model f is social functioning on psychological distress). Therefore, the effects of self-stigma on QoL can be seen as being divided into five parts: (1) a direct effect from self-stigma; (2) an indirect effect through psychological distress; (3) an indirect effect through social functioning; (4) an indirect effect through psychological distress, followed by social functioning; and (5) an indirect effect through social functioning, followed by psychological distress. Moreover, the effect of self-stigma on social functioning can be examined using: (1) a direct effect from self-stigma; and (2) an indirect effect through psychological distress. Furthermore, the effect of self-stigma on psychological distress can be examined using: (1) a direct effect from self-stigma; and (2) an indirect effect through social functioning. Comparing the five parts with our models, Models e and f can be decomposed into four parts (parts 1 to 4 for Model e; parts 1 to 3 and 5 for Model f); Model d can be decomposed into three parts (i.e., parts 1–3); Models b and c can be decomposed into two parts (parts 1 and 2 for Model b; parts 1 and 3 for Model c). In addition, Model a only contained the first part.

In all of the linear models, four important confounders (age, gender, educational year, and the presence of Hepatitis C virus) on QoL were included. We did not use any interaction or quadratic terms in the linear SEM models because we did not assume any moderation effects. Additionally, all the linear models treated QoL as a latent concept that consisted of four domains (physical, psychological, social, and environment) first. Afterward, the same five models were examined again for each QoL domain. That is, the independent variables, mediators, and confounders were the same with the changes in dependent variables. The reason to treat the QoL as a latent concept in the beginning is that we want to assess the overall health condition of our studied sample before investigating their specific QoL.

The mediated effects were investigated using bootstrap methods [51]: a total of 1000 bootstrap samples were performed using a 95% confidence interval, which did not contain zero, to determine an existing indirect effect [52]. In addition, we used an χ^2^ test, normed χ^2^ (i.e., χ^2^ divided by degree of freedom), comparative fit index (CFI), root mean square error of approximation (RMSEA), and standardized root mean square residual (SRMR) to decide whether the data fit well with each proposed model. A nonsignificant χ^2^ test; normed χ^2^ < 3; CFI > 0.9; RMSEA and SRMR < 0.08 indicated a satisfactory fit [53,54].

## 3. Results

Table 1 describes the demographics, clinical characteristics, and the scores in the self-stigma, QoL, psychological distress, and social functioning. In brief, the mean (±SD) age of the 250 participants was 45.12 ± 7.49 years, and nearly 90% were male (*n* = 224).

All the linear models had significant χ^2^ tests (*p* < 0.001 for all); however, other fit indices including the normed χ^2^ value, CFI, RMSEA, and SRMR were satisfactory or nearly satisfactory (Table 2). Moreover, Model f cannot be identified in our SEM analyses. Therefore, we could not describe any results for Model f. Nevertheless, Model a demonstrated that self-stigma negatively impacted the QoL of patients in MMT. Model b additionally indicated that the impacts of self-stigma on QoL were mediated by psychological distress with the indirect effects of psychological distress confirmed by 1000 bootstraps (Table 3). Model c indicated that social functioning also had negative impacts on the QoL of patients in MMT; however, there were no mediated effects demonstrated for self-stigma on QoL. Also, self-stigma was not shown to have any direct impact on social functioning (Table 3). Models d and e also demonstrated that the effects of self-stigma on QoL were mediated by psychological distress, as self-stigma directly influenced psychological distress but not social functioning (Table 3). 

We further compared Models d to e, and found that Model e outperformed Model d in all fit indices (Table 2). Moreover, the χ^2^ difference test showed that Model e was significantly improved (Δχ^2^ = 27.867, Δ*df* = 1, *p* < 0.001). We further calculated the proportions of the mediated effects, and the results showed that psychological distress was the primary mediator that accounted for about 70 to 75% of the mediated effects between self-stigma and QoL (Table 3). Moreover, results of the mediation models for each QoL domain were demonstrated in the Appendix tables (Please see Appendix A Table A1 for the summary of the fit indices; Appendix A Table A2 for the findings in physical QoL; Appendix A Table A3 for the findings in psychological QoL; Appendix A Table A4 for the findings in social QoL; and Appendix A Table A5 for the findings in environment QoL).

## 4. Discussion

The results of this study extend knowledge of the effects of self-stigma from people with severe mental illness to its effects on substance users. Corresponding to the negative effects of self-stigma on QoL in people with severe mental illness [24,25], similar results were found in our sample of patients in MMT. Our results further demonstrated that the effects of self-stigma were diminished when using psychological distress as a mediator; while effects existed when using social functioning as a mediator. Our findings, which corroborate with studies on healthy workers [28] and psychiatric outpatients [33], showed that the two mediators, psychological distress and social functioning, impact the QoL of patients in MMT. The effects of self-stigma on psychological distress in this study also agree with the results of other studies on people with mental illness [17,30]. Unlike the reported effects of self-stigma on social functioning [31], however, our findings showed that self-stigma did not have direct impacts on social functioning but rather had indirect effects through the mediator of psychological distress. The above results are somewhat in agreement with the findings of Mersh et al. [32], suggesting that the effects of self-stigma on social functioning diminished after controlling for symptom severity, a factor highly correlated with psychological distress [55]. In other words, psychological distress can moderate or mediate the effects of self-stigma on social functioning. That is, when people with high self-stigma simultaneously suffer from psychological distress, they might have worsened social functioning. In contrast, if people with high self-stigma have intact mental health, they might not have impaired social functioning.

According to the fit indices of the five proposed models, Models a (Figure 1a) and c (Figure 1c) exhibited the best fit. However, we felt that the two linear models did not fully capture approaches examining the effects of self-stigma on QoL, as was the case in studies [17,30] revealing the critical role of psychological distress in people with mental illness. Although being inferior to Models a and c, the fit indices of Model e (Figure 1e) were considered satisfactory. Given Model e included more information than did Models a and c, and significantly outperformed Model d, we believe that Model e reflected the real mechanism more than Models a and c did. 

Through Model e, mental health professionals can become aware of the importance of reducing self-stigma for substance users because of the higher levels of self-stigma associated with high psychological distress, decreased social functioning, and impaired QoL. The aforementioned results signify the value of intervention programs for self-stigma reduction [14,19]. That is, reducing self-stigma may help patients in MMT improve their mental health and social functioning, and in turn, their QoL, although Model e did not support the direct effects of self-stigma on QoL. Nevertheless, clinicians should not ignore indirect effects. In addition to self-stigma, Model e revealed that dealing with psychological distress and enhancing social functioning are other possible interventions by which clinicians can improve the QoL of patients in MMT. As a result, when a healthcare provider wants to improve the QoL of a patient in MMT, programs on self-stigma reduction, social functioning enhancement, and psychological health improvement are all potential approaches. Future studies may want to explore effective treatment programs on self-stigma, social functioning, and psychological health among patients in MMT given the importance of self-stigma, social health, and psychosocial health [56,57,58]. 

Our results showed that the self-stigma of most heroin users in MMT was high and the findings agree with the research in studying self-stigma for substance users [21,59,60]. About four fifths of our participants had SSS-S scores above 2.5, a cutoff that suggests high levels of self-stigma [9]. Can and Tanrıverdi [21] used the Internalized Stigma of Mental Illness scale [61] and found that nearly 85% of the people with substance abuse disorder scored above 2.5. Keyes et al. [60] and Heeren et al. [59] also found that people with alcohol use disorder have high levels of self-stigma. Additionally, the percentage of high level of self-stigma was much higher than that found in people with other mental disorders as reported by Chang et al. [62], where 21–40% of people with schizophrenia, 30–44% of those with bipolar disorder, 14–32% of those with depressive disorder, and 2–11% of those with anxiety disorder exhibited high levels of self-stigma. 

Our research adds to the expanding stigma literature demonstrating the negative effects of self-stigma on QoL through psychological distress among heroin users. Future studies may use our findings as the breaking point to further study the roles of self-efficacy and self-esteem in the association between self-stigma and health. Other factors related to mental illness are also needed to be discussed, such as social support, psychoeducation (for patients, public, family, and healthcare providers), coping strategies, and comorbid conditions (e.g., depression and anxiety) [23]. Additionally, routine psychological assessment combined with psychiatric service would be helpful to reduce the severe stigma problems among heroin users seeking MMT because most MMT programs in Taiwan have been established in the psychiatric department of hospitals. Furthermore, our findings may support policy on mental health to focus on the reduction of self-stigma and improvement of QoL among heroin users. Therefore, future studies are needed to explore whether the aforementioned factors (i.e., social support, psychoeducation, coping strategies, and comorbid condition) are useful in reducing self-stigma and improving QoL. 

There are some limitations in the study. First, the instrument we used (i.e., SSS-S) was not specifically for heroin users (or those in MMT). Instead, the SSS-S is an instrument widely used for people with different conditions [13], based on its replaceable terms for describing stigmatized populations. Therefore, the self-stigma we measured may not be as sensitive as instruments designed specifically for heroin users (or those in MMT) [16]. However, using the SSS-S has the advantage of comparing populations and the feasibility of comparing different populations. Second, following the first limitation, the psychometric properties of the SSS-S have never been examined using a sample with opioid use disorders. Therefore, the self-stigma as measured in this study may not be reliable or valid. However, we consider that this is not a serious problem because we examined the internal consistency of the SSS-S in this study, and the reliability results were satisfactory. Similarly, the WHOQOL-BREF used in this study is not a heroin-specific instrument on QoL. Therefore, the QoL measures in this study might not be sensitive to reflect the symptoms of heroin dependency. Third, the representativeness of our sample is restricted because we only recruited heroin users in MMT from central and southern Taiwan with a convenience sampling method. Therefore, it should be cautioned that our results may have limited generalizability. Fourth, we did not include people with other types of mental illness; therefore, we were unable to provide the direct evidence that people receiving MMT have significantly higher self-stigma than people with other mental diseases. Lastly and the most importantly, given that the data were collected in a cross-sectional design, our findings cannot be generalized to any causal relationship. Therefore, future studies using longitudinal design are needed to corroborate our findings. Specifically, in our cross-sectional design, Model f (Figure 1f) cannot be identified (i.e., our data could not fit with this specific conceptual model); however, we cannot ensure whether the model is also unidentified using a longitudinal design. 

## 5. Conclusions

In conclusion, this study demonstrated a linear model describing the effects of self-stigma on QoL for patients in MMT. Psychological distress was an important mediator between self-stigma and QoL. Although social functioning was not a mediator, it had direct impacts on QoL. Given that QoL is an important health outcome for heroin users (and those in MMT) [63] and based on our findings, clinicians may want to reduce self-stigma, decrease psychological distress, and improve the social functioning of this population.

## Figures and Tables

**Table 1:** Demographics, clinical characteristics, self-stigma, quality of life (QoL), psychological distress, and social function of participants.

	*n* (%)	Mean (SD)
**Demographics**		
Age in year		45.12 (7.49)
30 or below	2 (0.8%)	
31–40	61 (24.4%)	
41–50	120 (48.0%)	
51–60	59 (23.6%)	
61 or above	8 (3.2%)	
Missing	0 (0.0%)	
Gender		
Male	224 (89.6%)	
Female	26 (10.4%)	
Missing	0 (0.0%)	
Educational level		
Junior high or below	159 (63.6%)	
Senior high or above	91 (36.4%)	
Missing	0 (0.0%)	
Marital status		
Currently married	58 (23.2%)	
Others	192 (76.8%)	
Missing	0 (0.0%)	
Full-time employment		
Yes	126 (50.4%)	
No	124 (49.6%)	
Missing	0 (0.0%)	
Monthly income		
Less than minimum monthly wages in Taiwan	96 (38.4%)	
Greater than minimum monthly wages in Taiwan	154 (61.6%)	
Missing	0 (0.0%)	
**Clinical characteristics**		
Onset age in year		25.92 (6.97)
20 or below	65 (26.0%)	
21–30	135 (54.0%)	
31–40	38 (15.2%)	
41–50	11 (4.4%)	
Missing	1 (0.4%)	
Human immunodeficiency virus (HIV)		
Yes	36 (14.4%)	
No	214 (85.6%)	
Missing	0 (0.0%)	
Hepatitis B virus (HBV)		
Yes	45 (18.0%)	
No	205 (82.0%)	
Missing	0 (0.0%)	
Hepatitis C virus (HCV)		
Yes	178 (71.2%)	
No	72 (28.8%)	
Missing	0 (0.0%)	
Diabetes mellitus (DM)		
Yes	5 (2.0%)	
No	245 (98.0%)	
Missing	0 (0.0%)	
Hypertension (HTN)		
Yes	9 (3.6%)	
No	241 (96.4%)	
Missing	0 (0.0%)	
Previous episodes of MMT		
0	71 (28.4%)	
1	119 (47.6%)	
2 and above	60 (24.0%)	
Missing	0 (0.0%)	
**Self-stigma**		2.90 (0.59)
**QoL**		
Physical domain		13.26 (2.81)
Psychological domain		11.70 (2.97)
Social domain		12.47 (2.78)
Environment domain		13.19 (2.73)
**Psychological distress**		10.05 (6.41)
**Social functioning**		15.52 (6.33)

MMT: methadone maintenance treatment.

**Table 2:** Model fit for the conceptual models (*N* = 250).

Model #	χ^2^ (*df*)	*p*-Value	χ^2^/*df*	CFI	RMSEA	SRMR
Model a	194.540 (109)	<0.001	1.79	0.956	0.056	0.059
Model b	699.606 (361)	<0.001	1.94	0.901	0.061	0.065
Model c	207.475 (124)	<0.001	1.67	0.957	0.052	0.058
Model d	725.721 (387)	<0.001	1.88	0.902	0.059	0.064
Model e	697.854 (386)	<0.001	1.81	0.910	0.057	0.063

*df*: degree of freedom; CFI: comparative fit index; RMSEA: root mean square error of approximations; SRMR: standardized root mean square residual. Model a: Self-stigma on quality of life (QoL); Model b: Self-stigma on QoL mediated by psychological distress; Model c: Self-stigma on QoL mediated by social function; Model d: Self-stigma on QoL mediated by psychological distress and social function; Model e: Self-stigma on QoL mediated by psychological distress and social function, psychological distress on QoL mediated by social function. All the models were adjusted for the covariates of age, gender, educational years, and presence of hepatitis C virus (HCV).

**Table 3:** Direct and indirect effects of self-stigma on quality of life (QoL) using 1000 bootstraps (*N* = 250).

Model #	Coefficient (SE)/ *p*-Value (Lower Limit, Upper Limit)				% of Indirect Effects
	Self-Stigma	Psychological Distress	Social Function	Psychological Distress and Social Function	
**Direct effect**					
Model a	−1.967 (0.543)/ 0.002 (−3.11, −0.99)	--	--	--	--
Model b	−0.477 (0.321)/ 0.14 (−1.11, 0.15)	−3.098 (0.316)/ 0.001 (−3.72, −2.48)	--	--	--
Model c	−1.889 (0.492)/ 0.002 (−2.98, −1.06)	--	−0.143 (0.023)/ 0.002 (−0.19, −0.10)	--	--
Model d	−0.561 (0.341)/ 0.097 (−1.25, 0.12)	−2.886 (0.318)/ 0.001 (−3.59, −2.33)	−0.046 (0.019)/ 0.02 (−0.08, −0.007)	--	--
Model e	−0.594 (0.343)/ 0.07 (−1.26, 0.07)	−3.017 (0.322)/ 0.001 (−3.72, −2.47)	−0.049 (0.020)/ 0.01 (−0.09, −0.01)	--	--
**Indirect effect**					
Model b	--	−1.546 (0.421)/ 0.001 (−2.60, −0.91)	--	--	76.4%
Model c	--	--	−0.117 (0.150)/ 0.40 (−0.45, 0.17)	--	5.8%
Model d	--	−1.440 (0.231)/ <0.001 (−1.89, −0.99)	−0.038 (0.531)/ 0.94 (−1.08, 1.00)	--	72.5%
Model e	--	−1.406 (0.229)/ <0.001 (−1.85, −0.96)	−0.070 (0.524)/ 0.89 (−1.10, 0.96)	−0.106 (0.302)/ 0.73 (−0.70, 0.49)	70.9%

Model a: Self-stigma on quality of life (QoL); Model b: Self-stigma on QoL mediated by psychological distress; Model c: Self-stigma on QoL mediated by social function; Model d: Self-stigma on QoL mediated by psychological distress and social function; Model e: Self-stigma on QoL mediated by psychological distress and social function, psychological distress on QoL mediated by social function.

## Appendix A

**Table A1:** Model fit for the conceptual models in each domain of quality of life domains (*N* = 250).

Model #	χ^2^ (*df*)	*p*-Value	χ^2^/*df*	CFI	RMSEA	SRMR
**Physical**						
Model a	135.402 (68)	<0.001	1.991	0.946	0.063	0.061
Model b	562.358 (284)	<0.001	1.980	0.897	0.063	0.066
Model c	141.007 (80)	<0.001	1.763	0.952	0.055	0.059
Model d	582.013 (307)	<0.001	1.896	0.899	0.060	0.065
Model e	549.917 (306)	<0.001	1.797	0.911	0.057	0.065
**Psychological**						
Model a	135.413 (68)	<0.001	1.991	0.946	0.063	0.062
Model b	553.593 (284)	<0.001	1.949	0.899	0.062	0.067
Model c	142.267 (80)	<0.001	1.778	0.951	0.056	0.060
Model d	577.475 (307)	<0.001	1.881	0.900	0.059	0.066
Model e	552.040 (306)	<0.001	1.804	0.909	0.057	0.065
**Social**						
Model a	143.848 (68)	<0.001	2.115	0.939	0.067	0.062
Model b	551.124 (284)	<0.001	1.941	0.898	0.061	0.066
Model c	150.978 (80)	<0.001	1.887	0.944	0.060	0.060
Model d	574.387 (307)	<0.001	1.871	0.899	0.059	0.065
Model e	541.471 (306)	<0.001	1.770	0.911	0.056	0.064
**Environment**						
Model a	144.120 (68)	<0.001	2.119	0.939	0.067	0.064
Model b	561.155 (284)	<0.001	1.976	0.895	0.063	0.067
Model c	149.879 (80)	<0.001	1.873	0.945	0.059	0.061
Model d	582.081 (307)	<0.001	1.896	0.897	0.060	0.066
Model e	551.384 (306)	<0.001	1.802	0.908	0.057	0.065

**Table A2:** Direct and indirect effects of self-stigma on physical domain of quality of life (QoL) using 1000 bootstraps (*N* = 250).

Model #	Coefficient (SE)/ *p*-Value (Lower Limit, Upper Limit)				% of Indirect Effects
	Self-Stigma	Psychological Distress	Social Function	Psychological Distress and Social Function	
**Direct effect**					
Model a	−2.013 (0.559)/ 0.002 (−3.21, −1.04)	--	--	--	--
Model b	−0.349 (0.383)/ 0.38 (−1.07, 0.44)	−3.427 (0.351)/ 0.002 (−4.18, −2.83)	--	--	--
Model c	−1.909 (0.490)/ 0.002 (−3.00, −1.09)	--	−0.163 (0.024)/ 0.002 (−0.21, −0.12)	--	--
Model d	−0.445 (0.364)/ 0.26 (−1.08, 0.34)	−3.163 (0.352)/ 0.001 (−3.92, −2.58)	−0.054 (0.022)/ 0.01 (−0.10, −0.008)	--	--
Model e	−0.468 (0.360)/ 0.25 (−1.08, 0.33)	−3.350 (0.353)/ 0.001 (−4.14, −2.75)	−0.056 (0.023)/ 0.01 (−0.10, −0.01)	--	--
**Indirect effect**					
Model a	--	−1.678 (0.459)/ 0.001 (−2.89, −0.98)	--	--	82.8%
Model b	--	--	−0.133 (0.169)/ 0.41 (−0.50, 0.20)	--	6.5%
Model c	--	−1.550 (0.247)/ <0.001 (−2.03, −1.07)	−0.044 (0.532)/ 0.93 (−1.09, 1.00)	--	78.2%
Model d	--	−1.524 (0.244)/ <0.001 (−2.00, −1.05)	−0.079 (0.524)/ 0.88 (−1.11, 0.95)	−0.120 (0.307)/ 0.70 (−0.72, 0.48)	77.0%

**Table A3:** Direct and indirect effects of self-stigma on psychological domain of quality of life (QoL) using 1000 bootstraps (*N* = 250).

Model #	Coefficient (SE)/ *p*-Value (Lower Limit, Upper Limit)				% of Indirect Effects
	Self-Stigma	Psychological Distress	Social Function	Psychological Distress and Social Function	
**Direct effect**					
Model a	−2.326 (0.683)/ 0.003 (−3.69, −1.01)	--	--	--	--
Model b	−0.647 (0.494)/ 0.23 (−1.61, 0.35)	−3.341 (0.372)/ 0.002 (−4.11, −2.69)	--	--	--
Model c	−2.232 (0.647)/ 0.003 (−3.58, −1.04)	--	−0.148 (0.026)/ 0.002 (−0.20, −0.10)	--	--
Model d	−0.725 (0.341)/ 0.15 (−1.71, 0.23)	−3.147 (0.318)/ 0.002 (−3.92, −2.49)	−0.039 (0.019)/ 0.09 (−0.09, 0.007)	--	--
Model e	−0.784 (0.343)/ 0.13 (−1.79, 0.21)	−3.239 (0.322)/ 0.002 (−4.05, −2.56)	−0.044 (0.020)/ 0.06 (−0.10, 0.003)	--	--
**Indirect effect**					
Model a	--	−1.689 (0.448)/ 0.001 (−2.92, −1.04)	--	--	72.3%
Model b	--	--	−0.121 (0.156)/ 0.41 (−0.48, 0.17)	--	5.1%
Model c	--	−1.589 (0.259)/ <0.001 (−2.10, −1.08)	−0.032 (0.533)/ 0.95 (−1.08, 1.01)	--	69.1%
Model d	--	−1.526 (0.319)/ <0.001 (−2.15, −0.90)	−0.063 (0.527)/ 0.90 (−1.10, 0.97)	−0.085 (0.305)/ 0.78 (−0.68, 0.51)	66.6%

**Table A4:** Direct and indirect effects of self-stigma on social domain of quality of life (QoL) using 1000 bootstraps (*N* = 250).

Model #	Coefficient (SE)/ *p*-Value (Lower Limit, Upper Limit)				% of Indirect Effects
	Self-Stigma	Psychological Distress	Social Function	Psychological Distress and Social Function	
**Direct effect**					
Model a	−2.139 (0.579)/ 0.002 (−3.53, −1.18)	--	--	--	--
Model b	−0.831 (0.448)/ 0.050 (−1.83, 0.000)	−2.716 (0.366)/ 0.002 (−3.48, −2.02)	--	--	--
Model c	−2.048 (0.530)/ 0.001 (−3.37, −1.22)	--	−0.142 (0.027)/ 0.001 (−0.20, −0.09)	--	--
Model d	−0.937 (0.341)/ 0.02 (−1.85, −0.14)	−2.417 (0.318)/ 0.002 (−3.20, −1.76)	−0.060 (0.019)/ 0.02 (−0.11, −0.008)	--	--
Model e	−0.959 (0.433)/ 0.02 (−1.90, −0.21)	−2.580 (0.380)/ 0.002 (−3.36, −1.87)	−0.062 (0.027)/ 0.02 (−0.11, −0.10)	--	--
**Indirect effect**					
Model a	--	−1.317 (0.364)/ 0.001 (−2.23, −0.77)	--	--	61.3%
Model b	--	--	−0.116 (0.150)/ 0.40 (−0.45, 0.17)	--	5.4%
Model c	--	−1.175 (0.252)/ <0.001 (−1.67, −0.68)	−0.049 (0.532)/ 0.93 (−1.09, 0.99)	--	56.6%
Model d	--	-1.151 (0.256)/ <0.001 (−1.65, −0.65)	−0.087 (0.407)/ 0.83 (−0.88, 0.71)	−0.133 (0.315)/ 0.67 (−0.75, 0.48)	55.5%

**Table A5:** Direct and indirect effects of self-stigma on environment domain of quality of life (QoL) using 1000 bootstraps (*N* = 250).

Model #	Coefficient (SE)/ *p*-Value (Lower Limit, Upper Limit)				% of Indirect Effects
	Self-Stigma	Psychological Distress	Social Function	Psychological Distress and Social Function	
**Direct effect**					
Model a	−1.523 (0.546)/ 0.002 (−2.74, −0.64)	--	--	--	--
Model b	−0.168 (0.401)/ 0.71 (−0.94, 0.69)	−2.792 (0.326)/ 0.002 (−3.48, −2.17)	--	--	--
Model c	−1.444 (0.507)/ 0.002 (−2.59, −0.59)	--	−0.125 (0.025)/ 0.002 (−0.17, −0.07)	--	--
Model d	−0.232 (0.341)/ 0.59 (−0.99, 0.60)	−2.613 (0.318)/ 0.001 (−3.36, −1.97)	−0.035 (0.019)/ 0.16 (−0.08, 0.01)	--	--
Model e	−0.273 (0.343)/ 0.55 (−1.02, 0.58)	−2.748 (0.322)/ 0.002 (−3.51, −2.07)	−0.038 (0.020)/ 0.14 (−0.09, 0.01)	--	--
**Indirect effect**					
Model a	--	−1.362 (0.399)/ 0.002 (−2.37, −0.78)	--	--	89.0%
Model b	--	--	−0.103 (0.135)/ 0.37 (−0.42, 0.14)	--	6.7%
Model c	--	−1.278 (0.243)/ <0.001 (−1.75, −0.80)	−0.028 (0.534)/ 0.96 (−1.07, 1.02)	--	84.9%
Model d	--	−1.234 (0.247)/ <0.001 (−1.72, −0.75)	−0.053 (0.525)/ 0.92 (−1.08, 0.98)	−0.082 (0.312)/ 0.79 (−0.69, 0.53)	82.2%

## References

[1] Goffman E. (1963). Stigma: Notes on the Management of Spoiled Identity.

[2] Livingston J.D., Boyd J.E. (2010). Correlates and consequences of internalized stigma for people living with mental illness: A systematic review and meta-analysis. Soc. Sci. Med..

[3] Brohan E., Slade M., Clement S., Thornicroft G. (2010). Experiences of mental illness stigma, prejudice and discrimination: A review of measures. BMC Health Serv. Res..

[4] Corrigan P.W., Rao D. (2012). On the self-stigma of mental illness: Stages, disclosure, and strategies for change. Can. J. Psychiatry.

[5] Mehta S.I., Farina A. (1988). Associative stigma: Perceptions of the difficulties of college-aged children of stigmatized fathers. J. Soc. Clin. Psychol..

[6] Chang C.-C., Su J.-A., Tsai C.-S., Yen C.-F., Liu J.-H., Lin C.-Y. (2015). Rasch analysis suggested three unidimensional domains for Affiliate Stigma Scale: Additional psychometric evaluation. J. Clin. Epidemiol..

[7] Mak W.W.S., Cheung R.Y.M. (2008). Affiliate Stigma Among Caregivers of People with Intellectual Disability or Mental Illness. J. Appl. Res. Intellect. Disabil..

[8] Link B.G., Phelan J.C. (2001). Conceptualizing Stigma. Annu. Rev. Sociol..

[9] Boyd J.E., Adler E.P., Otilingam P.G., Peters T. (2014). Internalized Stigma of Mental Illness (ISMI) scale: A multinational review. Compr. Psychiatry.

[10] Chang C.-C., Wu T.-H., Chen C.-Y., Wang J.-D., Lin C.-Y. (2014). Psychometric evaluation of the internalized stigma of mental illness scale for patients with mental illnesses: Measurement invariance across time. PLoS ONE.

[11] Corrigan P.W., Michaels P.J., Vega E., Gause M., Watson A.C., Rüsch N. (2012). Self-stigma of mental illness scale—Short form: Reliability and validity. Psychiatry Res..

[12] Damghanian M., Alijanzadeh M. (2018). Theory of planned behavior, self-stigma, and perceived barriers explains the behavior of seeking mental health services for people at risk of affective disorders. Soc. Health Behav..

[13] Mak W.W.S., Cheung R.Y.M. (2010). Self-stigma among concealable minorities in Hong Kong: Conceptualization and unified measurement. Am. J. Orthopsychiatry.

[14] Luoma J.B., Kohlenberg B.S., Hayes S.C., Bunting K., Rye A.K. (2008). Reducing self-stigma in substance abuse through acceptance and commitment therapy: Model, manual development, and pilot outcomes. Addict. Res. Theory.

[15] Livingston J.D., Milne T., Fang M.L., Amari E. (2012). The effectiveness of interventions for reducing stigma related to substance use disorders: A systematic review. Addiction.

[16] Luoma J.B., Nobles R.H., Drake C.E., Hayes S.C., O’Hair A., Fletcher L., Kohlenberg B.S. (2013). Self-stigma in substance abuse: Development of a new measure. J. Psychopathol. Behav. Assess..

[17] Lin C.-Y., Chang C.-C., Wu T.-H., Wang J.-D. (2016). Dynamic changes of self-stigma, quality of life, somatic complaints, and depression among people with schizophrenia: A pilot study applying kernel smoothers. Stigma Health.

[18] Yen C.-F., Chen C.-C., Lee Y., Tang T.-C., Ko C.-H., Yen J.-Y. (2009). Association between quality of life and self-stigma, insight, and adverse effects of medication in patients with depressive disorders. Depress. Anxiety.

[19] Luoma J.B., Kohlenberg B.S., Hayes S.C., Fletcher L. (2012). Slow and steady wins the race: A randomized clinical trial of acceptance and commitment therapy targeting shame in substance use disorders. J. Consult. Clin. Psychol..

[20] Lin C.-Y., Strong C., Scott A.J., Broström A., Pakpour A.H., Webb T.L. (2018). A cluster randomized controlled trial of a theory-based sleep hygiene intervention for adolescents. Sleep.

[21] Can G., Tanrıverdi D. (2015). Social functioning and internalized stigma in individuals diagnosed with substance use disorder. Arch. Psychiatr. Nurs..

[22] Link B.G., Struening E.L., Rahav M., Phelan J.C., Nuttbrock L. (1997). On stigma and its consequences: Evidence from a longitudinal study of men with dual diagnoses of mental illness and substance abuse. J. Health Soc. Behav..

[23] Choo C.C., Chew P.K.H., Ho C.S., Ho R.C. (2019). Quality of life in patients with a major mental disorder in Singapore. Front. Psychiatry.

[24] Fung K.M.T., Tsang H.W.H., Corrigan P.W., Lam C.S., Cheung W., Cheng W. (2007). Measuring self-stigma of mental illness in China and its implications for recovery. Int. J. Soc. Psychiatry.

[25] Mashiach-Eizenberg M., Hasson-Ohayon I., Yanos P.T., Lysaker P.H., Roe D. (2013). Internalized stigma and quality of life among persons with severe mental illness: The mediating roles of self-esteem and hope. Psychiatry Res..

[26] Huang W.-Y., Chen S.-P., Pakpour A.H., Lin C.-Y. (2018). The mediation role of self-esteem for self-stigma on quality of life for people with schizophrenia: A retrospectively longitudinal study. J. Pac. Rim Psychol..

[27] Lin C.-Y. (2018). Comparing quality of life instruments: Sizing them up versus pediatric quality of life inventory and Kid-KINDL. Soc. Health Behav..

[28] Lu I.-C., Yen Jean M.-C., Lei S.-M., Cheng H.-H., Wang J.-D. (2011). BSRS-5 (5-item Brief Symptom Rating Scale) scores affect every aspect of quality of life measured by WHOQOL-BREF in healthy workers. Qual. Life Res..

[29] Pakpour A.H., Chen C.-Y., Lin C.-Y., Strong C., Tsai M.-C., Lin Y.-C. (2019). The relationship between children’s overweight and quality of life: A comparison of Sizing Me Up, PedsQL and Kid-KINDL. Int. J. Clin. Health Psychol..

[30] Yanos P.T., Roe D., Markus K., Lysaker P.H. (2008). Pathways between internalized stigma and outcomes related to recovery in schizophrenia spectrum disorders. Psychiatr. Serv..

[31] Lysaker P.H., Davis L.W., Warman D.M., Strasburger A., Beattie N. (2007). Stigma, social function and symptoms in schizophrenia and schizoaffective disorder: Associations across 6 months. Psychiatry Res..

[32] Mersh L., Jones F., Oliver J. (2015). Mindfulness, self-stigma and social functioning in first episode psychosis: A brief report. Psychosis.

[33] Trompenaars F.J., Masthoff E.D., Van Heck G.L., De Vries J., Hodiamont P.P. (2007). Relationships between social functioning and quality of life in a population of Dutch adult psychiatric outpatients. Int. J. Soc. Psychiatry.

[34] Hirschfeld R.M., Montgomery S.A., Keller M.B., Kasper S., Schatzberg A.F., Möller H.J., Healy D., Baldwin D., Humble M., Versiani M. (2000). Social functioning in depression: A review. J. Clin. Psychiatry.

[35] Chan K.K.S., Mak W.W.S. (2014). The mediating role of self-stigma and unmet needs on the recovery of people with schizophrenia living in the community. Qual. Life Res..

[36] Vauth R., Kleim B., Wirtz M., Corrigan P.W. (2007). Self-efficacy and empowerment as outcomes of self-stigmatizing and coping in schizophrenia. Psychiatry Res..

[37] Chang K.-C., Loh E.-W., Tang H.-P., Chen C.-M., Huang C.-J., Lan T.-H., Chiang M.-T., Kuo S.-W., Chiu H.-J. (2010). Disparity between heroin addicts enrolled in maintenance treatment and detoxification treatment and its implication. Asian J. Psychiatr..

[38] Chiang S.-C., Chan H.-Y., Chen C.-H., Sun H.-J., Chang H.-J., Chen W.J., Lin S.-K., Chen C.-K. (2006). Recidivism among male subjects incarcerated for illicit drug use in Taiwan. Psychiatry Clin. Neurosci..

[39] Stuart H. (2019). Managing the stigma of opioid use. Healthc. Manag. Forum.

[40] Chen Y.-M.A., Kuo S.H.-S. (2007). HIV-1 in Taiwan. Lancet.

[41] Lee C.T.C., Chen V.C.H., Tan H.K.L., Chou S.-Y., Wu K.-H., Chan C.-H., Gossop M. (2013). Suicide and other-cause mortality among heroin users in Taiwan: A prospective study. Addict. Behav..

[42] Wu T.-H., Chang C.-C., Chen C.-Y., Wang J.-D., Lin C.-Y. (2015). Further psychometric evaluation of the Self-Stigma Scale-Short: Measurement invariance across mental illness and gender. PLoS ONE.

[43] Darke S., Hall W., Wodak A., Heather N., Ward J. (1992). Development and validation of a multi-dimensional instrument for assessing outcome of treatment among opiate users: The Opiate Treatment Index. Br. J. Addict..

[44] Goldberg D.P., Hillier V.F. (1979). A scaled version of the General Health Questionnaire. Psychol. Med..

[45] Chan D.W., Chan T.S. (1983). Reliability, validity and the structure of the General Health Questionnaire in a Chinese context. Psychol. Med..

[46] Chou S.Y., Chan H.Y. (2015). The reliability and validity of the Modified Chinese Version of the Opiate Treatment Index. Taiwan J. Psychiatry.

[47] Chen C.-Y., Ting S.-Y., Tan H.K.-L., Yang M.-C. (2012). A multilevel analysis of regional and individual effects on methadone maintenance treatment in Taiwan. Value Health.

[48] Yao G., Chung C.-W., Yu C.-F., Wang J.-D. (2002). Development and verification of validity and reliability of the WHOQOL-BREF Taiwan version. J. Formos. Med. Assoc..

[49] Lin C.-Y., Hwang J.-S., Wang W.-C., Lai W.-W., Su W.-C., Wu T.-Y., Yao G., Wang J.-D. (2019). Psychometric evaluation of the WHOQOL-BREF, Taiwan version, across five kinds of Taiwanese cancer survivors: Rasch analysis and confirmatory factor analysis. J. Formos. Med. Assoc..

[50] MacKinnon D.P. (2008). Introduction to Statistical Mediation Analysis.

[51] Fritz M.S., Mackinnon D.P. (2007). Required sample size to detect the mediated effect. Psychol. Sci..

[52] Lin C.-Y., Tsai M.-C. (2016). Effects of family context on adolescents’ psychological problems: Moderated by pubertal timing, and mediated by self-esteem and interpersonal relationships. Appl. Res. Qual. Life.

[53] Lin C.-Y., Luh W.-M., Cheng C.-P., Yang A.-L., Ma H.-I. (2014). Evaluating the wording effect and psychometric properties of the Kid-KINDL: Using the multitrait-multimethod approach. Eur. J. Psychol. Assess..

[54] Tsai M.-C., Strong C., Lin C.-Y. (2015). Effects of pubertal timing on deviant behaviors in Taiwan: A longitudinal analysis of 7th- to 12th-grade adolescents. J. Adolesc..

[55] Phillips M.R. (2009). Is distress a symptom of mental disorders, a marker of impairment, both or neither?. World Psychiatry.

[56] Chan Y., Chan Y.Y., Cheng S.L., Chow M.Y., Tsang Y.W., Lee C., Lin C.-Y. (2017). Investigating quality of life and self-stigma in Hong Kong children with specific learning disabilities. Res. Dev. Disabil..

[57] Cheng M.Y., Wang S.-M., Lam Y.Y., Luk H.T., Man Y.C., Lin C.-Y. (2018). The relationships between weight bias, perceived weight stigma, eating behavior, and psychological distress among undergraduate students in Hong Kong. J. Nerv. Ment. Dis..

[58] Pakpour A.H., Lin C.-Y., Alimoradi Z. (2018). Social health and behavior needs more opportunity to be discussed. Soc. Health Behav..

[59] Heeren T., Edwards E.M., Dennis J.M., Rodkin S., Hingson R.W., Rosenbloom D.L. (2008). A comparison of results from an alcohol survey of a prerecruited Internet panel and the National Epidemiologic Survey on Alcohol and Related Conditions. Alcohol. Clin. Exp. Res..

[60] Keyes K.M., Hatzenbuehler M.L., McLaughlin K.A., Link B., Olfson M., Grant B.F., Hasin D. (2010). Stigma and treatment for alcohol disorders in the United States. Am. J. Epidemiol..

[61] Ritsher J.B., Otilingam P.G., Grajales M. (2003). Internalized stigma of mental illness: Psychometric properties of a new measure. Psychiatry Res..

[62] Chang C.-C., Wu T.-H., Chen C.-Y., Lin C.-Y. (2016). Comparing self-stigma between people with different mental disorders in Taiwan. J. Nerv. Ment. Dis..

[63] Lin C.-Y., Chang K.-C., Wang J.-D., Lee L.J.-H. (2016). Quality of life and its determinants for heroin addicts receiving a methadone maintenance program: Comparison with matched referents from the general population. J. Formos. Med. Assoc..

